# Skin Grafting

**Published:** 1905-08-12

**Authors:** 


### Editor's Letter-Box.

### SKIN GRAFTING.

Mr. A. Young writes: My attention has been drawn to a notice in your paper *re* my article on Skin Grafting in International Clinics (Vol. I., Series XV.). While thanking you for the kind reference you make, may I draw your attention to the fact that a sentence in the last paragraph is not sufficiently clear to avoid misinterpretation? The sentence is as follows:—" Sometimes the grafts do not 'take' at all, while quite commonly, after growing well for a time, they ulcerate, and show other evidences of deficient vitality." I take it that this statement is either made of Thiersch's grafts, of which the sentence immediately preceding speaks, or it is an expression of your opinion regarding grafts made as I suggest. If the former, there is a lack of clearness in the reference; if the latter, I can only say that my experience is quite in the opposite direction, as I have pointed out in my paper. On page 99 of the "Clinics" it is stated that, "in my experience, Thiersch grafts are extremely uncertain, while grafts applied on this plan, however, almost always take." Very seldom, in my experience, have grafts, once taken, undergone subsequent degenerative change.

.˙. We regret that there should seem any ambiguity in the article to which Mr. Young refers. The sentence which he quotes has reference, of course, to Thiersch's grafts, and is in continuation of the sentence which immediately precedes it. Degenerative changes in Thiersch's grafts which have once taken are not rare, for example, when the grafts have been applied to the leg in the treatment of chronic ulceration.—*Editor of* The Hospital.

